# Neuromodulation of sensory networks in monkey brain by focused ultrasound with MRI guidance and detection

**DOI:** 10.1038/s41598-018-26287-7

**Published:** 2018-05-22

**Authors:** Pai-Feng Yang, M. Anthony Phipps, Allen T. Newton, Vandiver Chaplin, John C. Gore, Charles F. Caskey, Li Min Chen

**Affiliations:** 1 Department of Radiology and Radiological Sciences, Vanderbilt University Medical Center, Nashville, TN, USA; 2 Vanderbilt University Institute of Imaging Science, Nashville, TN, USA

## Abstract

Focused ultrasound (FUS) has gained recognition as a technique for non-invasive neuromodulation with high spatial precision and the ability to both excite and inhibit neural activity. Here we demonstrate that MRI-guided FUS is capable of exciting precise targets within areas 3a/3b in the monkey brain, causing downstream activations in off-target somatosensory and associated brain regions which are simultaneously detected by functional MRI. The similarity between natural tactile stimulation-and FUS- evoked fMRI activation patterns suggests that FUS likely can excite populations of neurons and produce associated spiking activities that may be subsequently transmitted to other functionally related touch regions. The across-region differences in fMRI signal changes relative to area 3a/3b between tactile and FUS conditions also indicate that FUS modulated the tactile network differently. The significantly faster rising (>1 sec) fMRI signals elicited by direct FUS stimulation at the targeted cortical region suggest that a different neural hemodynamic coupling mechanism may be involved in generating fMRI signals. This is the first demonstration of imaging neural excitation effects of FUS with BOLD fMRI on a specific functional circuit in non-human primates.

## Introduction

Regions and circuits within a healthy brain work together in a coordinated fashion; dysfunction in such networks underlie several neurological disorders^1–7^. Identifying how different regions of the brain, many of which are often functionally independent, work together to execute specific cognitive tasks and maintain proper function remains a major challenge. In particular, identifying the causal relationships between nodes within and among different networks is crucial for understanding their operation and functions. Brain stimulation, specifically the manipulation of activity within circuits in both positive (excitation) and negative (inhibition) directions, can be a powerful approach to directly correlate changes in neural circuits with functional and behavioral readouts and establish the causal relationships between brain regions. To date, the most widely used stimulation methods for studying human brain function include transcranial electrical stimulation^8–11^, intracranial electrical stimulation^12–15^, deep brain stimulation^16–18^, and transcranial magnetic stimulation^4,19–22^. Among these methods, only transcranial electrical and magnetic stimulation are non-invasive and can localize primarily superficial brain structures on the centimeter scale, while the others are highly invasive, requiring surgical placements of electrodes into the brain. FUS offers a practical neuromodulation method that has ideal characteristics: (1) it is noninvasive, (2) offers millimeter scale spatial localization, (3) can access all parts of the brain easily, (4) is compatible with accurate imaging of brain activity, and (5) can potentially modulate brain activity in both excitatory and inhibitory directions.

Image guidance not only improves the spatial precision of positioning the US focal region but also enables simultaneous characterization of both local (at target) and associated circuit (off-target) neural responses. Other investigators have recognized the importance of image-guidance in FUS neuromodulation, and typically use stereotactic frames or optical tracking systems to localize the focus in pre-acquired images^23,24^. We have established similar procedures in our laboratory, which permit optically-guided tracking of the ultrasound transducer and projection of the acoustic beam into MR planning images. Our approach allows real-time beam localization along with functional mapping based on BOLD (blood oxygenation level dependent) fMRI. Combining FUS with MRI has potential to be a very powerful technique to interrogate neural circuits.

In the present study, we aimed to address several challenges and questions about the application of FUS neuromodulation of the non-human primate brain using MRI-guided FUS at 7 T. We chose to study the monkey somatosensory system because it has been well characterized using fMRI and intracranial electrophysiology as well as invasive and post-mortem studies^25,26^. We have developed robust fMRI paradigms that allow us to reliably map brain touch circuits in non-human primates^27–32^. We first established that a FUS transducer could be implemented in a 7 T human scanner, with an optical tracking system to guide transducer placement. We then investigated whether long blocks containing FUS bursts could induce detectable BOLD signals, and how these compared to BOLD effects induced by peripheral cutaneous tactile stimulation. An overarching goal was to evaluate whether FUS can selectively activate brain circuits in a functionally specific manner. We showed that FUS evoked robust BOLD activations at the target and off-target regions within the touch functional circuit, and that FUS-induced BOLD signal changes are generally similar to those evoked by tactile stimulation, but they can be much stronger and rise faster.

## Results

### fMRI mapping of tactile stimuli-evoked responses

**Figure 1:**
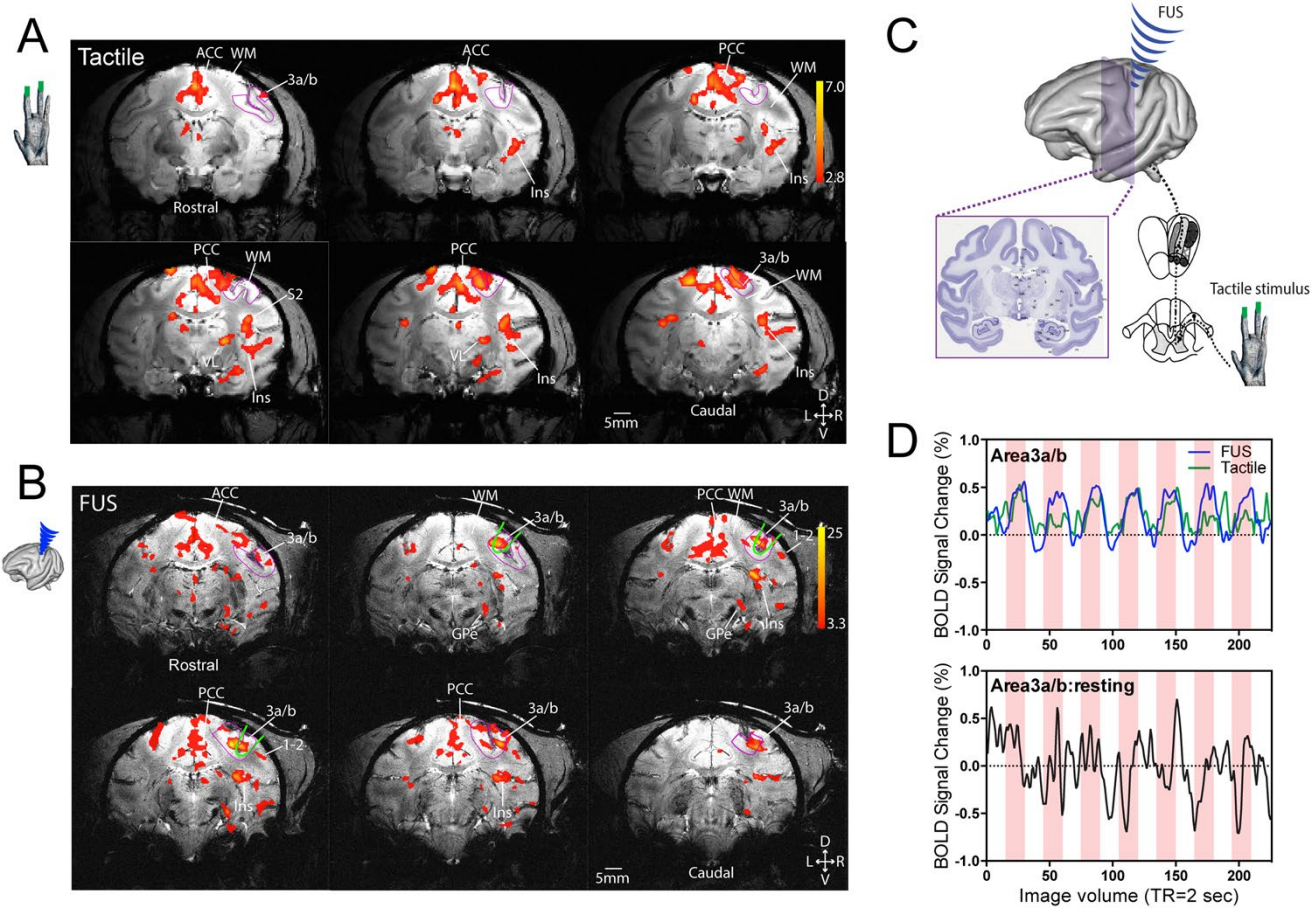
Comparison of tactile stimulus-evoked and Focused ultrasound stimulation (FUS)-elicited fMRI BOLD activations in the macaque brain. (**A**) Representative single run coronal fMRI activation maps evoked by 8 Hz stimulation of distal finger pads of digits 2&3 of left hand of subject 1. Activation maps are thresholded at t > 2.8, *p* = 0.005, *q* = 0.012, FDR corrected. (**B**) Representative single run coronal fMRI activation maps evoked by FUS stimulation of the areas 3a/3b region of right hemisphere of subject 2. Activation maps are thresholded at t > 3.3, *p* = 0.001, *q* = 0.003, FDR corrected. Green oval outlines indicate the focus (25% power) locations of the ultrasound beam. Magenta outlines show the areas 3a/3b region. WM: white matter. ACC: anterior cingulate cortex. PCC: posterior cingulate cortex. Ins: insular cortex. 3a/b: areas 3a and 3b. 1–2: areas 1 and 2. VL: thalamic ventral-lateral nucleus. GPe: globus pallidus. D: dorsal. V: ventral. L: left. R: right. Six coronal images are arranged from rostral to caudal direction (top left to bottom right). (**C**) A diagram shows the two experimental conditions examined in the study: peripheral tactile stimulation of digits and direct cortical stimulation with FUS. (**D**) BOLD signal time courses derived from areas 3a/3b voxels during tactile and ultrasound stimulation (top panel) and at rest (without stimulation, bottom panel). Light pink background strips indicate the stimulus duration, they are for reference only in the resting state panel.

Vibrotactile stimulation produced by an oscillating vertical probe indentation (with 0.48 mm displacement) on distal digits of the hand is transmitted through the spinal dorsal column pathway and cuneate nucleus to the ventral-posterior-lateral (VPL) nucleus of the thalamus, and then reaches the primary (areas 3b and 1 of S1) and secondary (S2) somatosensory as well as posterior insular (Ins) cortices of the non-human primate brain. We used 8 Hz vibrotactile stimuli because it is a subtle and natural cutaneous stimulus that elicits robust touch sensation in humans and activates many cortical and subcortical brain regions, including S1 and S2 in the non-human primate^27,28,33–35^. We have previously demonstrated that many of the areas responding to such a stimulus are detectable as fMRI activations at ultra-high field (9.4 T) in the brains of squirrel monkeys under light anesthesia^30,31^ and also show correlated signal variations in a resting state, reflecting their functional connectivity^36,37^. Extending our previous observations in squirrel monkeys, Fig. 1A shows the same 8 Hz tactile stimuli-evoked fMRI activations in contralateral areas 3a/3b and areas 1/2 of S1, S2, posterior and anterior Insula (Ins), VPL nucleus, and posterior and anterior cingulate cortices (PCC and ACC) at 7 T in the macaque brain. The BOLD time course extracted from activated areas 3a/3b region exhibited robust (~0.5%) stimulus-evoked signal changes (see the green line in Fig. 1D, top panel). In contrast, the BOLD signals obtained during a baseline state (i.e. without stimulation) showed only low-level random fluctuations (see the black line in Fig. 1D, bottom panel). Similar tactile stimulus-evoked activation patterns were observed repeatedly across imaging sessions and animals.

### FUS stimulation of areas 3a/3b of S1 cortex elicited fMRI activations in touch related regions

**Figure 2:**
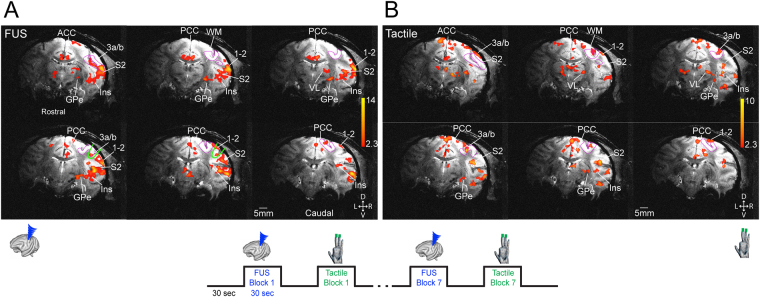
Interleaved presentation of FUS and tactile stimulation evoked similar fMRI activation patterns. (**A**) A single run BOLD fMRI activation map evoked by FUS of area 3a/3b region of right hemisphere. (**B**) Tactile stimulus-evoked fMRI activation maps. Activation maps are thresholded at t > 2.3, *p* < 0.02, *q* = 0.03, FDR corrected. Magenta outlines show the areas 3a/3b region. D: dorsal. V: ventral. L: left. R: right. D: dorsal. Bottom insert illustrates the stimulus (FUS and tactile) presentation protocol. Six rostral to caudal coronal images are arranged from top left to bottom right. V: ventral. L: left. R: right.

To evaluate the effects of FUS pulses, we chose a functionally defined touch region in areas 3a/3b as a target area because it is the first cortical relay station within touch circuits, and direct stimulation of this region would likely activate at least part of the touch circuit. Functional circuits identified by tactile stimulation can thus serve as a reference for the functional relevance and neural basis of FUS effects. We therefore directly targeted the FUS beam to the area 3a/3b activation focus identified by fMRI response to tactile stimulation in each animal, and insonated the region with an identical 30 sec on/off stimulation paradigm (see 50% FUS power targets (green outlines) in Fig. 1B images). FUS elicited fMRI activations in several tactile regions (compare Figs 1B and 2). The BOLD time courses extracted from areas 3a/3b during FUS stimulation exhibited very similar signal increases as those during tactile stimulation (compare blue and green lines in Fig. 1D). To illustrate the stimulation effects of FUS, Fig. 2 shows the FUS (A) and tactile stimulation-evoked (B) fMRI activations maps obtained within the same interleaved fMRI data acquisition run (see the insert for the stimulus presentation paradigm). This finding shows that block-design FUS pulses elicited similar hemodynamic responses as natural and subtle vibration tactile stimulation at the targeted region.

### Distinct response profiles of touch versus FUS in a subset of brain network regions

**Figure 3:**
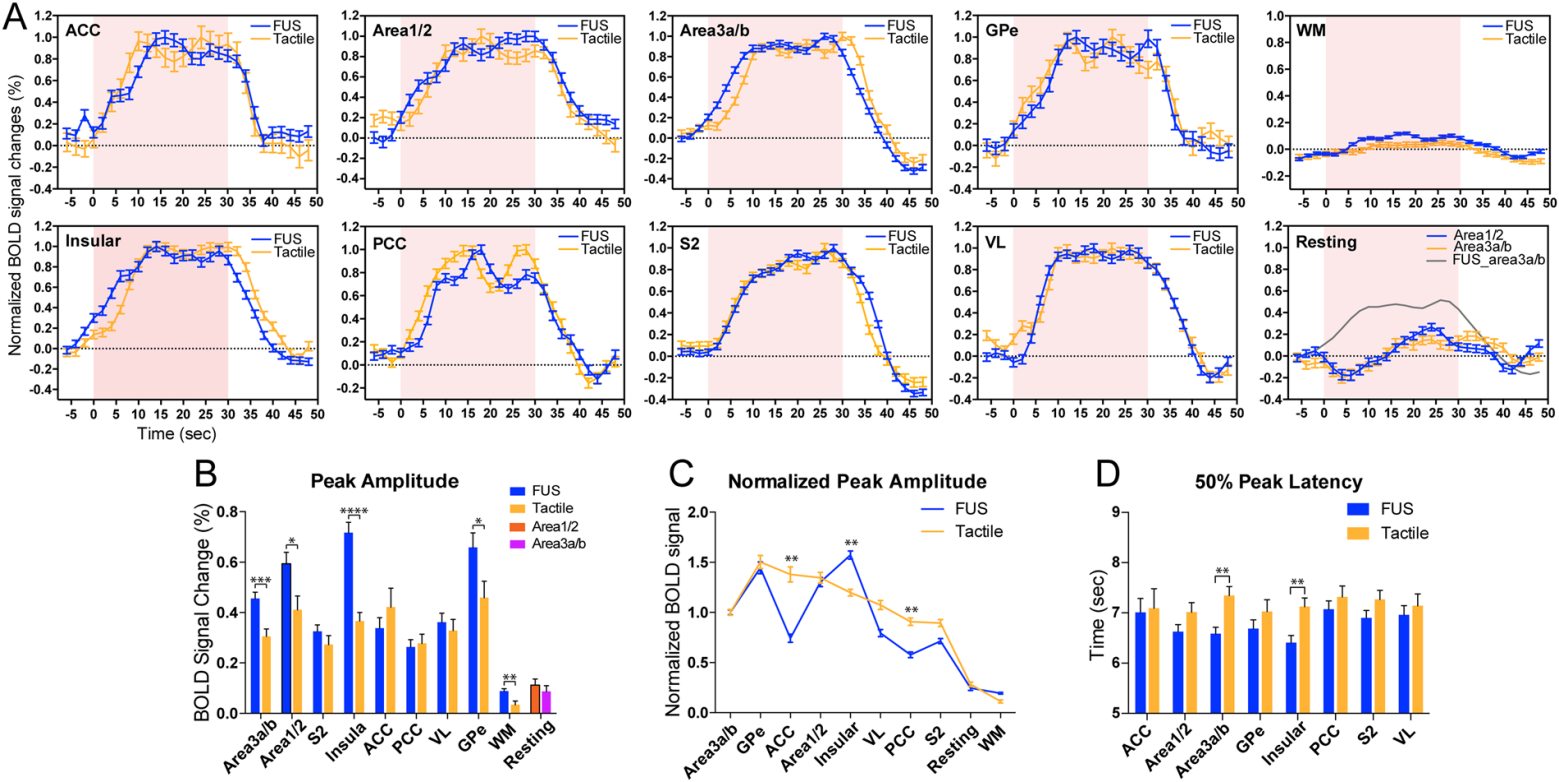
Group comparison of BOLD time courses, signal amplitude, rising slopes, and response latency during tactile versus FUS stimulation in different brain regions. (**A**) Normalized BOLD time courses (peak is considered as 1) derived from eight activated regions (by either tactile stimulation or FUS) and two control conditions: white matter (WM) region and at resting state. Shadows indicate the standard error distribution. Light pink background strips indicate the 30-sec stimulation period. (**B**) Comparison of absolute peak BOLD% signal changes evoked by tactile stimulation versus FUS in each individual ROI. *p < 0.05, **p < 0.01, ***p < 0.005, ****p < 0.001, unpaired t-test. (**C**) Rearranged normalized BOLD signal changes plot (signal of areas 3a/3b is considered as 1) from high to low for tactile stimulation and corresponding values for FUS. *p < 0.05, **p < 0.01, nonparametric Mann-Whitney test. (**D**) Direct comparisons of 50% peak latency in each ROI. **p < 0.01, unpaired t-test. FMRI data from a total of 13 tactile and 19 FUS runs from six imaging sessions in two animals were included in the group quantification.

Based on the hierarchical organization of the touch circuit, we speculated that direct stimulation of areas 3a/3b, bypassing the thalamus, likely alters the information flow between touch regions within the somatosensory functional circuit. To test this hypothesis, we quantified and compared% BOLD signal changes and their temporal features at ten selected ROIs (eight activated and two control regions) and calculated group averages (n = 13 tactile runs and n = 19 FUS runs). Figure 3A shows the normalized (to the peak) mean (±SEM) event-related BOLD time courses in response to either tactile or FUS stimulation. Their overall temporal profiles were comparable. Percentage BOLD signal increases were generally stronger during FUS (up to 0.8%) than tactile stimulation (up to 0.5%) (Fig. 3B). The peak signal amplitudes were significantly higher in areas 3a/3b, areas 1/2, and insular cortices (*p* < 0.005, *p* < 0.05, and *p* < 0.001, unpaired t-test; compare blue with green columns in Fig. 3D). As a control, signals derived from white matter (WM) voxels were very weak (<0.1%). Areas 3a/3b BOLD signals obtained during resting state showed only asynchronous fluctuations around baseline (last panel in Fig. 3A and last column group in Fig. 3B).

Further analysis of the time courses revealed that the BOLD signal changes in relative to that of area 3a/3b (defined as 1) varied markedly across regions between tactile stimulation and FUS conditions (Fig. 3C). For example, the BOLD signal amplitude in ACC was lower in FUS but much higher in tactile stimulation condition. Similar differences were observed in three other regions of insular, VL and PCC (Fig. 3C). Moreover, the FUS-evoked fMRI signals rose faster than those by tactile stimulation in areas 3a/3b and insular cortex, with significantly shorter (~1 sec) 50% peak latency (Fig. 3D).

## Discussion

The potential of neuromodulation by FUS has previously been illustrated in human and *in vivo* animal studies^38,39^; however, the mechanisms underlying the effects of FUS on neurons have not been established. For example, in small animals, physical movements have been induced by insonifying the motor cortex, while visual evoked potentials have been inhibited by insonifying the visual areas of the brain for multiple seconds^40,41^. A prior human showed that low amplitude, transcranial FUS focused on the S1 region of the somatosensory cortex inhibited electroencephalogram (EEG) signals evoked by stimulating the median nerve^42^. Another report more recently demonstrated that US neuromodulation of the primary somatosensory cortex (S1) elicited specific sensations. The ability of FUS to modulate neural activity in both positive and negative directions has been repeatedly validated. However, how these FUS neuromodulation effects are accomplished remains unclear, and their impacts on networks and mediation via neural circuits have not been explored.

Using BOLD fMRI signal as a readout, we show here that blocks of FUS pulses not only excite the targeted cortical region, but also elicit BOLD activations in spatially distant regions that are a part of the cutaneous touch network and beyond. FUS-induced effects have been previously reported in the visual system of behaving macaques, which one may expect to elicit neural activity and a network-scale BOLD response^24,43^. Our study is the first evidence of imaging FUS’s excitation effects with BOLD fMRI not only at the target but also as a cause of modulation in downstream brain regions. The similarity between the stimulus-evoked touch and FUS fMRI responses demonstrate the targeted stimulation propagates throughout a specific brain functional circuit (the touch circuit in our case) without the primary input from touch receptors, depicting the underlying functional connectivity. Another significant finding is that blocks of FUS induced hemodynamic BOLD responses similar to external stimuli, but these were significantly stronger and faster rising in the targeted areas 3a/3b, its neighboring areas 1/2, and posterior insular cortex, compared to their responses to the specific cutaneous tactile stimulation used here. Two main features of the BOLD responses support the conclusion that FUS-induced effects are neural based and circuit specific. First, the overall shape of BOLD signal changes evoked by long FUS blocks is very similar to the typical hemodynamic signal changes evoked by natural tactile stimulation, which are known to be related to increases in spiking activities and local field potentials in areas 3b and 1 of S1, as well as S2, within the monkey somatosensory system^32,36^. Second, the FUS-induced responses in these regions differed in magnitude and temporal features between regions. BOLD signals detected at the targeted and neighboring regions were twice as strong and twice as fast to rise compared to those of off-target regions. We interpret the responses of the off-target regions as neural-based and activated via inter-regional connections. Very weak MRI signals in adjacent white matter and in a resting state support our conclusion that FUS-induced effects are region- and circuit-selective, rather than a global, non-neural effect in the brain.

Although the specific neural effects induced by FUS remain to be determined, existing evidence suggest that FUS pulses may excite (i.e., generate neuronal spikes), inhibit or modulate (change neuron excitability) neural activity^44,45^. The detection of BOLD signal increases at both targeted and distant off-target regions led us to speculate that FUS likely excited neurons and resulted in spiking activities at the target location, which subsequently excited those functionally inter-connected brain regions within the circuit. This finding is of significance for interpreting FUS effects on behavioral changes because it localizes the neuronal effects of FUS to the circuit level, particularly at the frequency and parameters we used in the current study. The present study demonstrates the feasibility and sensitivity of BOLD signals for detecting neuronal effects of FUS. Whether the precise mechanism of FUS-induced BOLD signals are neuronal versus vascular remains to be further investigated, but the different temporal responses compared to tactile stimulation may reflect differences in the balance of changes in blood oxygenation versus flow between the two conditions.

The specific mechanism of FUS neuromodulation remains unknown, but information can be gained from our experiments about the role and magnitude of acoustic radiation force and temperature. Our results do not suggest that radiation force plays a direct role in the observed BOLD fMRI signal. As a net displacement over a region, we would not expect micron-scale displacements due to acoustic radiation force to generate an effect on the BOLD signal. If the pulse compressed the vascular geometry over a wide region, it may be feasible that such a small displacement could modify the BOLD response, but we believe this is unlikely in our study since the magnitude of displacement is very low. Prior studies at 220 kHz have reported displacements as high as 10 μm at acoustic powers multiple orders of magnitude greater than those used in our study at 250 kHz, so we may expect the radiation force to be less than 1 μm^46^. Regardless, the BOLD response occurs seconds after neural activation has begun and the tissue relaxes on the millisecond scale, so we conclude that interactions between the radiation force and BOLD fMRI would be indirect if present. Temperature rise during FUS stimulation is a potential mechanism, so we tracked temperature in all experiments with MR thermometry. The average temperature increase measured in regions of interest (ROIs) in the ultrasound focus during ultrasound blocks was zero mean (0.00 +/− 0.15 °C) and the maximum temperature increase detected in the sonicated brain ROI across all ultrasound blocks was 0.5 °C. These temperature increases are consistent with bioheat estimates at the power used (data not shown).

Finally, it is worth noting that even though the neural excitatory effects of FUS were apparent in our experiments, the precise stimulation locus and spatial distribution of the effects remain to be further validated with real-time MRI measures, such as acoustic radiation force imaging (ARFI)^47–49^. Image-guided targeting of the FUS focus with optical tracking, as used here and by others^23^, is accurate to within ~3 mm but incurs a degree of variability in the final location of the core FUS region. Thus, we attribute the concurrent strong fMRI activations in areas 3a/3b, areas 1/2, and insular to this uncertainty. What’s more important is the fact that given all these variabilities, BOLD signal increases at the targeted areas 3a/3b and its immediate neighboring areas 1/2 and posterior insula were retained at the group level, across different scans and animals. Future real-time MRI monitoring of the FUS beam will provide more accurate and precise information about the final core targets.

## Methods

### Animal preparation

Two adult male macaque monkeys (*M. fascicularis*) were studied; each animal was scanned three times (a total of six sessions). Animals were initially sedated with ketamine hydrochloride (10 mg/kg) and atropine sulfate (0.05 mg/kg) and then anesthetized with isoflurane (1.0–1.5%) delivered over oxygen. After intubation, the animal was placed in a custom-designed MR stereotaxic frame with the head secured by ear bars, eye, and mouthpieces. During functional MRI data acquisition, animals were maintained at a light (0.85–1.0%) and stable level of anesthesia. A solution of 2.5% dextrose in saline solution was infused intravenously (3 ml/kg/h) to prevent dehydration. Animals were artificially ventilated throughout the experiment. Rectal temperature was maintained (SA Instruments) between 37.5 °C and 38.5 °C by means of a circulating water blanket. Heart rate and peripheral capillary oxygen saturation (SpO2; Nonin), respiration pattern and end-tidal CO_2_ (24–32 mmHg; SurgiVet) were continuously monitored and maintained during the entire procedure. All procedures were conducted in accordance with National Institutes of Health guidelines and were approved by the Institutional Animal Care and Use Committee of Vanderbilt University.

### Peripheral tactile stimulation protocol

The fingers were stabilized with modeling clay, palm side up, leaving the glabrous skin of the distal finger pads available for stimulation. Innocuous tactile stimuli were delivered at 8 Hz on the distal finger pads of D2 and D3 of the left hand in 0.44 mm vertical displacements via two rounded probes 2 mm in diameter driven by an S88 Grass stimulator. For each fMRI tactile stimulation run, 30-second duration blocks of tactile stimuli were repeated seven times with 30 second baseline periods in between. The probes were in contact with the skin of the digits during baseline periods.

### Localization of ultrasound beam and stimulation

**Figure 4:**
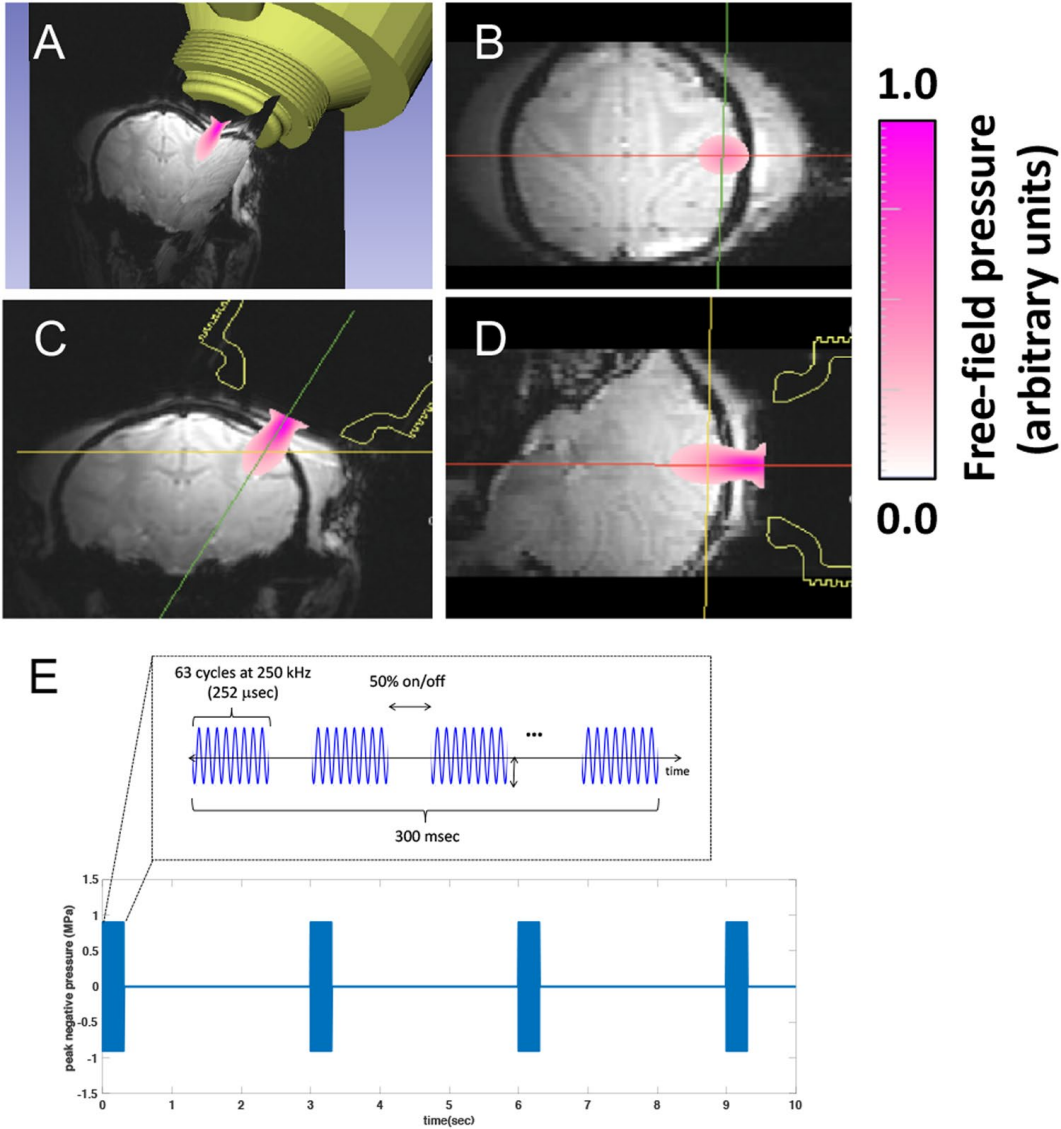
MRI guided FUS stimulation set up. (**A**) 3D view of the spatial relationships of the FUS transducer location, estimated beam path using the optically-guided real-time projection of the freefield beam, and the targeted brain region. (**B**–**D**) Overlaid free field FUS beam on MRI images on three different imaging planes (**B**: coronal, **C**: axial, **D**: sagittal). (**E**) PUS pulse design. Color bars represent the scaled pressure range.

A FUS transducer was positioned over the central sulcus, where the primary somatosensory cortex (S1) resides, on the right hemisphere ipsilateral to the tactile stimulation side of the left hand. Conducting gel was placed over the scalp to ensure adequate acoustic coupling. Six MRI visible fiducial markers (15 mm outer diameter) (MM3002, IZI Medical Products Owings Mills, MD) were placed one on each eyebar, one on each ear bar, and two on the side of the head that was not being sonicated. Tactile stimulus-evoked activation in area 3b/3a cortex, which was obtained from a prior imaging session, was used as a guide for aiming the FUS beam focus (see Fig. 4). The location and path of the FUS beam were estimated by optically tracking the transducer (NDI Polaris, Ontario, Canada) and registering to the imaging space via fiducials. To overlay the beam map, we collected a 3D acoustic map in the laboratory using an optically-tracked needle hydrophone in a water tank, with the tracked transducer coupled to the side. During stimulation experiments, the free field beam map was projected into co-registered MRI voxels, using the fiducial markers localized in both MRI and optically-tracked coordinates with 3D Slicer48. We note that the free field beam would be aberrated by the skull but that we expect this to be minimal with the current transducer geometry at 250 kHz based on prior simulations of comparable skulls. Average targeting error after image registration is estimated to be about 3 mm, and the registration method follows our prior work49.

### Ultrasound parameters and power

During fMRI data acquisition, FUS pulses were delivered in a block-based scheme identical to the tactile stimuli in seven 30 sec FUS on/off cycles (see Fig. 2 insert). The FUS transducer was driven by an arbitrary waveform generator (Agilent 33511B, Santa Clara, CA, USA) connected to an RF power amplifier (E&I A150, Rochester, NY, USA). A custom microcontroller program coordinated pulsing for tactile or FUS stimulation. During an “on” block, a 250 kHz, 300 msec pulse was emitted at 0.333 Hz from a spherically focused MR-compatible transducer (H115, Sonic Concepts, Bothell, WA) coupled via a custom Fomblin-filled cone (Fomblin Y LVAC 06/6, Solvay, Mt. Pleasant, TN). The Fomblin-filled cone was used to replace water to eliminate susceptibility artifacts and geometric distortions of MRI images due to ineffective shimming, since Fomblin contains no hydrogen. A single 300 msec pulse consisted of 63-cycle bursts repeated at 2 kHz (50% duty cycle–see Fig. 4E for FUS pulse design). The procedure resulted in 10 FUS bursts per “on” block.

The maximum free field pressure of the transducer was measured by placing a calibrated needle (for pressures lower than MI = 1.4, H-0400 Onda Corp.) or fiber optic hydrophone (at higher pressures, FOH, Precision Acoustics, Dorchester, UK) at the focus of the transducer and recording the peak negative pressure at different input amplitudes. Measurements were performed with both fomblin and water in the coupling cone and the peak pressure was within 10 percent for each of these materials at their respective foci. The highest free field peak negative pressure (PNP) used for neuromodulation was estimated to be 935 kPa which corresponds to a mechanical index (MI) of 1.87, a spatial peak pulse average (I_sppa_) of 29.5 W/cm^2^, and a spatial peak temporal average (I_spta_) of 1.34 W/cm^2^. We chose this pressure as an upper bound to elicit a maximal effect while remaining below the MI limit for body imaging (1.9) with ultrasound, while noting that these standards were established for short pulses and should not necessarily be considered as safety metrics for neuromodulation. The skull attenuates ultrasound so a lower pressure is expected inside the brain. To estimate how much the skull lowers the pressure, a piece of *ex vivo* macaque skull was placed underwater between the needle hydrophone and the water filled coupling cone of the transducer. Pressure measurements were made with the skull at five different orientations similar to those used in *in vivo* experiments. On average the maximum measured pressure behind the skull was 58% of the free-field beam during transcranial sonication. Using this as a basis for attenuation, we estimate ultrasound values in the brain during neuromodulation to be a PNP of 543 kPa, an MI of 1.08, an I_sppa_ of 9.9 W/cm^2^, and an I_spta_ of 0.452 W/cm^2^. When derated for the skull, these acoustic pressures are similar to those reported in Lee *et al*.^50^.

### MR thermometry

We calculated a phase image from the real and imaginary images acquired during every fMRI sequence. Using the phase information, we created a temperature map for each scan with the proton resonance frequency shift method with baseline correction^51–53^. We averaged 5 phase maps immediately prior to each task block to create a phase baseline for a given block and then computed the temperature change in each acquisition during the task relative to this baseline. An ROI was manually selected in the brain where we expected the FUS focus to be located based on optically tracked beam overlays. An additional ROI in the brain outside the FUS beam was selected. We then calculated the mean temperature change in the brain ROIs within and outside the expected FUS focus for each FUS and tactile block in all studies.

### MRI data acquisition and analyses

All MRI scans were performed on a 7 T Philips Achieva magnet with a customized surface transmit-receive coil (inner diameter = 6 cm) centered over the S1 and S2 cortices of the right hemisphere. Three types of MRI images were acquired: 3D T1-weighted high-resolution isotropic volume examination (THRIVE) images were acquired to localize the fiducial markers placed around the US probe for aligning and localizing the US beam to structural MRI images (TR = 1.89 ms, TR = 4 ms, flip angle 10°, NSA = 1) (Fig. 4A). A series of nine T2*-weighted multi-slice gradient echo high-resolution structural coronal images (TE = 10 ms, TR = 500 ms, 768 × 768 × 9 matrix, 0.104 × 0.104 × 1 mm^3^ voxel size) were also collected. FMRI data were acquired from the same slices using a single-shot gradient echo planar imaging (GE-EPI) sequence (TE = 16 ms, TR = 2 sec, 0.625 × 0.625 × 1 mm^3^ voxel size, 128 × 128 × 9 matrix, interleaved slices, linear K space filling). An extra navigator echo was collected with no phase encoding prior to the acquisition of the actual image data. This echo is used to correct for phase variations typically caused by motion. FMRI data with tactile stimulation of distal digits and/or FUS were collected with the same functional imaging acquisition parameters. To maximize the activation detection, two different stimulation presentation paradigms are used in fMRI data acquisition: single stimulation (tactile or FUS) and interleaved tactile and FUS conditions. Non-interleaved experiments were performed sequentially in the same imaging session and that anesthesia level was closely monitored and adjusted, so that the vital signals remained very stable (heart rate:160 ± 3; end tidal CO_2_: 34 ± 1, and pulse oxygenation: 99 ± 1%). In a typical single condition fMRI session (day), a total 6 to 10 tactile stimulation and US runs (225 volumes per run) were acquired. In a typical interleaved session, a total of 3 to 4 runs were acquired (435 volumes per run). A total of six imaging sessions (three sessions from each monkey) were included in the fMRI time course analysis.

### fMRI data analysis

#### Pre-processing

FMRI signals, acquired during tactile stimulation, FUS, or resting state, went through standard pre-processing steps of slice timing (3dTshift, AFNI) and 3-D motion correction (3dvolreg, AFNI), and then spatially smoothed using an isotropic Gaussian filter kernel with a full width at half maximum of 1 mm (3dmerge, AFNI). Functional EPI images were up-sampled from 0.625 × 0.625 × 1 mm^3^ to 0.312 × 0.312 × 1 mm^3^, and co-registered with corresponding T2*-weighted high-resolution anatomical images using a linear image registration tool (3dAllineate, AFNI) for display. The fMRI EPI data were temporally smoothed with a low-pass filter with cutoff frequency of 0.25 Hz (fslmaths, FSL).

#### FMRI activation maps

FMRI activation maps were created using a cross-correlation function between the signal time courses of each voxel and the boxcar predictor of the HRF convolved stimulus presentation paradigm (3dDeconvolve, AFNI). Activation was defined by voxels that exhibited significantly correlated BOLD signal changes (*p* < 0.02, *p* < 0.005 or *p* < 0.001, FDR corrected) and were organized in a minimum of five up-sampled continuous voxels (cluster size of 0.49 mm^3^). Thresholded fMRI activation maps (with statistical t-values, typically thresholded at t = 2.3 (*p* = 0.02), t = 2.8 (*p* = 0.005) or 3.3 (*p* = 0.001), FDR corrected) were spatially interpolated and then superimposed on the corresponding high-resolution T2*-weighted anatomical images.

### Quantification of time course of BOLD signal

Regions of interest (ROI)-based time course analyses were performed. Three types of voxels were defined: (1) cortical voxels activated by tactile or ultrasound stimulation, (2) white matter voxels, and (3) voxels showing apparent changes during resting state (i.e., runs without stimulation). We extracted the BOLD signal time courses from voxels with a maximal t-value > 2.8 within each ROI to quantify the amplitudes and temporal profiles of the BOLD signal changes to stimulation. White matter (WM) voxels were defined based on anatomical images. Resting state BOLD signal time courses were extracted from peak voxels in two regions (areas 3a/b and areas 1/2) that exhibited strong responses to FUS or tactile stimulation during resting state. All ROIs were defined using a macaque monkey brain atlas^54^. Each run contains 8 non-stimulus blocks (30 s) interleaved with 7 stimulus blocks (30 s). We calculated the time course of the percent signal change by subtracting the mean baseline signal prior to stimulus onset from the mean BOLD signals of three peak volumes during the stimulation period, and then dividing it by the baseline signal ((peak-baseline)/baseline%). The slope of the BOLD signal change was calculated between stimulus onset and 50% of peak response time points. BOLD peak amplitudes were calculated in each stimulus block from the 12 plateau image volumes during stimulus presentation. Measures were averaged across runs, scan sessions and animals, and were examined for statistical significance using an unpaired t-test method between tactile and US stimulation conditions. A *p* < 0.05 was considered statistically significant. ROI-based BOLD time course plots are presented as the mean ± standard error (SEM).
